# Association between Allogeneic Blood Transfusion and Wound Infection after Total Hip or Knee Arthroplasty: A Retrospective Case-Control Study

**DOI:** 10.7150/jbji.30636

**Published:** 2019-04-20

**Authors:** Ashish Taneja, Ahmed El-Bakoury, Hoa Khong, Pam Railton, Rajrishi Sharma, Kelly Dean Johnston, Shannon Puloski, Christopher Smith, James Powell

**Affiliations:** 1 Cumming School of Medicine, University of Calgary, 3330 Hospital Drive NW, Calgary, Alberta, T2N 4N1, Canada; 2 University of Alexandria, Egypt; 3 Alberta Bone and Joint Health Institute, 3280 Hospital Drive NW, Calgary, Alberta, T2N 4Z6, Canada; 4 Alberta Health Services, Calgary, Alberta, Canada; 5 McCaig Institute for Bone and Joint Health

**Keywords:** total hip replacement, total knee replacement, blood transfusion, infection

## Abstract

**Background:** To assess using a retrospective case control study, whether patients undergoing primary, elective total hip or knee arthroplasty who receive blood transfusion have a higher rate of post-operative infection compared to those who do not.

**Materials and Methods:** Data on elective primary total hip or knee arthroplasty patients, including patient characteristics, co-morbidities, type and duration of surgery, blood transfusion, deep and superficial infection was extracted from the Alberta Bone and Joint Health Institute (ABJHI). Logistic regression analysis was used to compare deep infection and superficial infection in blood-transfused and non-transfused cohorts.

**Results:** Of the 27892 patients identified, 3098 (11.1%) received blood transfusion (TKA 9.7%; THA 13.1%). Overall, the rate of superficial infection (SI) was 0.5% and deep infection (DI) was 1.1%. The infection rates in the transfused cohort were SI 1.0% and DI 1.6%, and in the non-transfused cohort were SI 0.5% and DI 1.0%. The transfused cohort had an increased risk of superficial infection (adjusted odds ratio (OR) 1.9 [95% CI 1.2-2.9, p-value 0.005]) as well as deep infection (adjusted OR 1.6 [95% CI 1.1-2.2, p-value 0.008]).

**Conclusion:** The odds of superficial and deep wound infection are significantly increased in primary, elective total hip and knee arthroplasty patients who receive blood transfusion compared to those who did not. This study can potentially help in reducing periprosthetic hip or knee infections.

## Introduction

Peri-prosthetic joint infections (PJIs) are the most disastrous complications of total hip or knee arthroplasty 1,2. Besides the increased morbidity and mortality for patients, these infections are a huge burden on healthcare resources^1^,^2^. A significant volume of research is being done in accurately diagnosing PJIs^3^,^4^. However, similar efforts are not being allocated to identify modifiable risk factors, which may help in preventing PJIs^5^. Allogeneic blood transfusion may be a potential risk factor for infection after total hip or knee arthroplasty^6^,^7^. With lack of an accepted universal protocol for post- operative blood transfusions, large variation is seen in the proportion of TJA patients receiving blood transfusion postoperatively 8,9,10,11,12. Substantial evidence exists showing that patients receiving allogeneic blood transfusion after TJA have an increased risk of prolonged length of hospital stay, 90-day mortality, respiratory and urinary tract infections, and wound healing disturbances 13,14, 15,16,17,11,12.

When examining the literature, there seems to be an association between allogenic blood transfusion and increased risk of infection^8^,^18^,^7^. However, there still remains some controversy within the literature that despite large database inquiry, for example the National Surgical Quality Improvement Program (NSQIP), there seems to be the lack of evidence supporting this association 19.

There are several studies examining the association between blood transfusion and infection 14,8,10. This study used a large database for a retrospective review of this problem.

The aim of our study was to analyze the association between allogenic blood transfusion after elective primary total hip or knee arthroplasty and rate of post-operative superficial and deep wound infection. Our hypothesis was that allogenic blood transfusion is associated with a higher rate of wound infection.

## Methods

We identified patients over 18 years of age who underwent primary, elective total hip and total knee arthroplasty (THA and TKA, respectively) in Alberta between April 1, 2012 and March 31, 2015. Exclusion criteria included patients undergoing surgery for revision, peri-prosthetic fractures and infection. Details on patient characteristics (age, gender), co-morbidities, type and duration of surgery, and use of blood transfusion were extracted from the ABJHI's data repository. Superficial and deep wound infections within six months of surgery were identified by Infection Control Specialists as part of the provincial surgical site infection surveillance program 20. Superficial infections include infections limited to the skin and subcutaneous tissue. Deep infections include both deep incisional and organ/space infections.

ABJHI is a non-profit organization that maintains a provincial musculoskeletal data repository used for quality improvement reporting and feedback. The repository prospectively captures data on all primary, elective TJA patients at 14 hospitals in the province of Alberta from various databases, including physician electronic medical record systems, operating room information systems, Alberta Health Services (AHS) Infection Prevention and Control, Discharge Abstract Database (DAD), National Ambulatory Care Reporting System (NACRS), and clinical risk grouper (CRG) data 21,22. DAD is a national database that captures administrative, clinical, and demographic information on every hospital discharge 23. NACRS is a national database that captures administrative, clinical, and demographic information on every day surgery, emergency department, and selects outpatient community-based clinic visits 24. CRG is a proprietary diagnostic-based risk-adjustment system licensed by the Alberta health authority from 3M Inc. to detect the presence of clinical risks using administrative data sets 25.

CRG data were used to detect the presence of 12 pre-surgery risk factors deemed by the Alberta Hip and Knee Arthroplasty Clinical Committee and the ABJHI to be highly relevant to arthroplasty outcomes (cardiac illness, chronic pulmonary condition, cancer, stroke, history of deep vein thrombosis or pulmonary embolus, chronic hepatic condition, chronic renal condition, diabetes with complications, obesity, dementia, and moderate to severe mental illness).

As part of the ongoing provincial surgical site infection (SSI) surveillance program, records on every primary, elective hip and knee arthroplasty were cross-linked to DAD and NACRS to identify any hospital readmission or ambulatory care visit within 6 months of surgery 20. Any such visit where select anti-infective therapies were administered was examined by an Infection Control Professional (ICP). ICPs used National Healthcare Safety Network SSI definitions to determine whether a patient met SSI criteria 26.

The provincial guidelines for blood transfusion were revised on January 1, 2015 to initially transfuse 1 unit instead of 2, although no change was made to the threshold at which transfusion was recommended (Table 7).

Descriptive analysis was performed on patient characteristics (age, sex, and pre-surgery risk factors), type and duration of surgery. Continuous variables were summarized as a mean ± standard deviation (SD) along with the range of values, while categorical variables were expressed as number and percentages. Un-paired t-test was used to compare continuous variables and Pearson's chi-square or Fisher's exact test was used to compare categorical variables.

Patients were stratified by age (“<70 years” and “≥70 years”), sex, number of pre-surgery risk factors (“none”, “one”, “two or more”), procedure type (“THA” and “TKA”), and procedure duration from skin incision to skin closure (“<100 minutes” and “≥100 minutes”) for both THA and TKA patients. Descriptive analysis was conducted to compare the transfused and non-transfused cohorts and to compare the deep and superficial infection cohorts to the non-infected cohorts. Multivariable logistic regression was used to calculate the odds of getting deep or superficial infection in the transfused and non-transfused cohorts after adjusting for age sex, pre-surgery risk factors, type and duration of procedure. Significance was set as 0.05. The area under the receiver operator characteristics (ROC) curve was calculated to estimate the predictive power of the multivariable regression model 27,28,29.

All statistical analyses were performed with STATA version 13 (STATA, College Station, Tex) software. All the data was de-identified by the ABJHI according to the Alberta health authority's de-identification standards, as a result consent was waived. This study was approved by our University's Research Ethics Board (REB).

## Results

27 892 patients were included in our study. 16 036 (57.5%) were female and the mean age was 66.3 + 10.4 years (Table 1). TKA was the most common procedure (16385; 58.7%) compared to THA (including hip resurfacing) (11507; 41.3%). In total, 14125 (50.6%) had no pre-surgery risk factors, 6802 (24.4%) had one risk factor and 6965 (25.0%) had two or more risk factors. Blood transfusion occurred in 3098 (11.1%) patients, with a significantly higher proportion of females in the transfused cohort (71.7%) compared to the non-transfused cohort (55.7%) (p-value<0.001) (Table 2). The likelihood of blood transfusion significantly increased with age (p-value <0.001) and for patients with two or more risk factors (p-value<0.001). Blood transfusion was significantly higher in THA patients compared to TKA (p-value<0.001) and when the duration of surgery was 100 minutes or longer (p-value<0.001).

The rate of deep infection for the cohort was 1.1% (n=305/27892) (Table 3). THA had a significantly higher rate of deep infection (1.4%; n=159/11507) compared to TKA (0.9%; n=146/16385) (p-value <0.001). Rate of superficial infection was 0.5% (n= 149 /27,892), with no significant difference between THA and TKA (p=0.68). Duration of surgery did not have any significant influence on the rate of deep infection (p-value=0.48) or superficial infection (p-value=0.31).

Transfused cohort had a significantly higher rate of deep infection (1.6%; n=51/3095) compared to the non-transfused cohort (1.0%; n=254/24797) (p-value= 0.002) with unadjusted odds ratio (OR) of 1.6 (95% CI: 1.2-2.2) (Table 4). Superficial infection showed a significant association with an infection rate of 1.0% 9 (n=31/3095) in the transfused cohort and 0.5% (n=118/24797) in the non-transfused cohort (p-value <0.001) with an unadjusted OR of 2.1 (95% CI: 1.4-3.1).

The results of multivariate logistic regression analysis show that there is a statistically significant positive association between deep infection and blood transfusion (adjusted OR 1.6; 95% CI: 1.1-2.2), and having two or more pre-surgery risk factors (adjusted OR 1.7; 95% CI 1.3-2.2) (Table 5). Moreover, a significant negative association between deep infection and TKA vs. THA (adjusted OR 0.6; 95% CI: 0.5-0.8), and female sex (adjusted OR 0.7; 95% CI: 0.5-0.8) is shown. There was no statistically significant relationship between deep infection and duration of procedure (adjusted OR 1.0; 95% CI: 0.7-1.5), age (adjusted OR 1.0; 95% CI: 0.8-1.3), and having one pre-surgery risk factor (adjusted OR 1.2; 95% CI: 0.9-1.6).

Similarly, there was a statistically significant association between superficial infection and blood transfusion (adjusted OR 1.9; 95% CI: 1.2-2.9), and having one (adjusted OR 2.3; 95% CI: 1.5-3.6) or two or more pre-surgery risk factors (adjusted OR 2.5; 95% CI: 1.6-3.8) (Table 6). There was no statistically significant relationship between superficial infection and procedure type (adjusted OR 1.1; 95% CI: 0.8-1.6), duration of procedure (adjusted OR 1.2; 95% CI: 0.7-2.0), age (adjusted OR 0.8; 95% CI 0.6-1.2) and sex (adjusted OR 1.1; 95% CI: 0.8-1.6). The deep infection and superficial infection multivariable regression models are estimated to have moderate predictive power with area under the ROC curve of 0.70 and 0.68, respectively.

## Discussion

We observed a transfusion rate of 11.1% in 27,892 patients, with the rate of blood transfusion decreasing over the three-year period. While this prevalence is high compared to current day standards, it is lower than that was reported in other published studies 8,10,19,30. We suspect this could be related to the growing awareness amongst surgeons about the possible complications of blood transfusion as well as more focus on pre-operative optimization of the patients and improved clinical practice related to blood conservation (e.g. increased use of tranexamic acid) 31.

Regarding both superficial and deep infections, data were captured for six months after the index surgery. The rate of deep infection was 1.1% in our cohort. The rate of deep infection in THA (1.4%) was significantly higher than TKA (0.9%) and duration of surgery did not significantly affect the rate of deep or superficial infections. A significant association was found between peri-operative allogeneic blood transfusion and superficial (adjusted OR 1.9) as well as deep infection (adjusted OR 1.6) after TJA. The rate of deep infection was found to be lower in TKA (adjusted OR 0.6) and females (adjusted OR 0.7) compared to THA and males, respectively. Pre-operative risk factors were found to have significant association with both deep and superficial infection.

Within the literature, some studies show no significant difference in the rate of wound infection after total hip or knee arthroplasty between patients who receive versus those that did not receive a blood transfusion. 10,19. On the other hand, few reports have shown positive association between allogenic blood transfusion and the increase in the incidence of SSI (Table 8).

Newman et al 10 in their study on 3352 patients undergoing total hip or knee arthroplasty did find higher rate of wound infection requiring reoperation in patients who received allogeneic blood transfusion, but could not find statistical significance after adjusting for other variables. They also found that autologous blood transfusion group had similar rate of infection when compared to the non-transfusion group. Hart et al 19 used NSQIP database to identify the prevalence, risk factors and 30-day complication rate for blood transfusion after total hip or knee arthroplasty. The cohort included 23024 patients (9362 THA and 13662 TKA). This study showed significant association between blood transfusion within 72 hours of surgery and deep infection within 30 days of index surgery on univariate analysis, however the association was not significant on multivariate analysis.

In contrast, evidence exists within the literature that allogeneic blood transfusion is associated with an increased length of hospital stay 14,15, increased risk of 90-day mortality^13^, increased risk of respiratory and urinary tract infections 14,16,17. However, the evidence is conflicting when it comes to the relationship between allogeneic blood transfusion and wound infection. Friedman et al 8 did a post hoc analysis of the pooled data of 12000 patients from the RECORD (Regulation of Coagulation in Orthopedic Surgery to Prevent Deep Venous Thrombosis and Pulmonary Embolism) study and found that there was a significant increase in any infection, lung infection with upper/lower respiratory tract infection and wound infection in elective total knee and hip arthroplasty patients receiving blood transfusion compared to those patients who received auto transfusion or no transfusion. Similar results were also obtained in an institution based study done by Frisch et al 30. They found a significantly higher rate of deep infection in the transfused group (2.4%) compared to the non-transfused group (0.5%) (p-value=0.01). Kim et al 18 performed a meta- analysis on 21,770 patients and found a significantly higher frequency of surgical-site infections based on pooled analysis using a random- effect model (2.88% on transfused and 1.74% non- transfused). However, this meta-analysis included six studies with only one having around 12000 patients and the rest were small studies having around 1000 patients included. Therefore, our study represents the largest study in the literature suggesting a significantly higher infection rate in patients who underwent allogeneic blood transfusion. Additionally, our study will strengthen the overall conclusion of future meta- analyses investigating this topic. Moreover, our current study supports the association of both superficial and deep infection after allogenic blood transfusion.

There was inconsistency of the criteria used to define SSI. In this study, we used the National Healthcare Safety Network criteria which were similar to Al Mustafa et al 32 who retrospectively reported on 3932 and found an increased incidence of SSI in patients having allogenic blood transfusion. Other studies, however, have used mainly reoperation and readmission for suspected infection as an indication for SSI 33. This inconsistency of the definition of SSI, obviously, could have had an implication on the final reported results.

The postoperative follow up periods reported in previous studies addressing the association between blood transfusion and SSI were variable (Table 8). Our follow up period was 6 months after the index procedure and this was longer than what was reported by Friedman et al 34 and Newman et al 33 (follow up of 1.5 and 3 months respectively). In spite of having smaller numbers of patients in their cohorts, other investigators included a postoperative follow up of 1 year 32, 35. These differences in the length of follow up, subsequently, can lead to different incidences of SSI as a short duration follow up can miss late-manifested low-grade infections.

One of the other factors that can be attributed to the variability of the incidence SSI is the type of the index surgical procedure whether it was a primary or revision arthroplasty. Similar to some other reports (table 8) 32, 33 we only included patients with primary hip and knee arthroplasty in this study trying to minimize the heterogenicity of the investigated cohort. Despite performing a separate statistical analysis for the revision and primary arthroplasty cases included in their study, Everhart et al 35 have reported a higher incidence of SSI. They have attributed this to the complexity of their cases as well as the more sensitive surveillance methods they have used to detect patients with SSI.

The exact cause for the association of blood transfusion to infection is unknown. Some speculation has suggested the possibility of transfusion-induced immunomodulation 6. Recent research suggests allogenic transfusion results in the release of IL4 and IL10, which causes suppression of Th1 response that is important in immune activity 7. Others suggest the storage of blood product releases suppressors from leukocytes including T-cell anergy 6. Yet another theory suggests apoptotic cells being immunosuppressive 37. Unfortunately, the exact mechanism of immunomodulation due to blood transfusion is unknown.

To our knowledge, this study is amongst the largest in the published literature. Also, our study is more generalizable as it includes patients operated at all surgical centers in Alberta and is less biased by single individual or single institutional policies and practices. Because this is a provincial registry it is much less likely that a patient would develop an infection and not be identified. A vast majority of patients would have had subsequent care in the province in follow up. The study does have limitations. Being a retrospective and observational study, causality between blood transfusion and wound infections cannot be proven. The number of blood unit transfused was not available. Despite our best efforts to control for confounding variables, (including but not limited to the threshold for transfusion is lower in patients with increased number of comorbidities) there is always a possibility of bias and confounding variables 38,39. However, efforts to mitigate bias included the use of a high-quality database, large sample size and an analysis of other potential confounding variables including age, gender, number of medical comorbidities, THA or TKA and prolonged surgery. True causation can only be determined using a prospective randomized clinical trial, however, such a study design may be unethical.

The results of this study further support the association between allogenic blood transfusion and superficial and deep PJI. This helps realize the need to establish new policies targeted at better pre-operative patient optimization and improvement in pre-operative hemoglobin levels using simple measures like iron supplements or Erythropoietin injections 30,40,41. There is a potential for further research to establish specific guidelines for allogeneic blood transfusion and the threshold of post-operative hemoglobin that warrants blood transfusion.

## Conclusion

Patients receiving blood transfusion after an elective, primary THA and TKA have 1.6 times higher odds of getting deep infection and 1.9 times higher odds of getting superficial infection compared to those who do not receive blood transfusion.

## Figures and Tables

**Table 1:** Description of study cohort.

Study cohort	27892
**Age (years) (mean ± SD)**	66.3 ± 10.4 (min=18, max=103)
**Female (%)**	16036 (57.5)
**Pre-surgery risk factors:**	
None (%)	14125 (50.6)
One (%)	6802 (24.4)
Two or more (%)	6965 (25)
**Procedure type:**	
TKA (%)	16385 (58.7)
THA, incl. hip resurfacing (%)	11507 (41.3)
**Procedure duration (no duration data on 2176 patients):**	
<100 mins (%)	23144 (90)
≥100 mins (%)	2572 (10)
**Transfused (%)**	3098 (11.1)
**Superficial infection (%)**	149 (0.5)
**Deep infection (%)**	305 (1.1)

**Table 2:** Descriptive analysis of transfused versus non-transfused cohort (unadjusted).

	Transfused N=3095 (11.1%)	Non-Transfused N=24797 (88.9%)	p-value
**Age (years)** **(mean ± SD)**	70.8 + 11.3 (min=18, max=96)	65.7 + 10.2 (min=18, max=103)	<0.001*
**Female (%)**	2220 (71.7)	13816 (55.7)	<0.001**
**Pre-surgery risk factors:**			
None (%)	1020 (32.9)	13105 (52.9)	<0.001**
One (%)	851 (27.5)	5951 (24.0)	
Two or More (%)	1224 (39.5)	5741 (23.1)	
**Procedure Type:**			
TKA (%)	1583 (51.1)	14802 (59.7)	<0.001**
THA, incl. hip resurfacing (%)	1512 (48.8)	9995 (40.3)	
**Procedure Duration:**			
<100 mins (%)	2355 (83.2)	20789 (90.8)	<0.001**
≥100 mins (%)	476 (16.8)	2096 (9.2)	

* t test for mean comparison. ** Chi-square test

**Table 3:** Descriptive analysis of deep infection and superficial infection cohorts (unadjusted)

	Deep Infection			Superficial Infection		
	Yes N=305 (1.1%)	No N=27587 (98.9%)	p-value	Yes N=149 (0.5%)	No N=27743 (99.5%)	p-value
**Age(years) (mean ± SD)**	65.7 + 11.8 (min=20, max=92)	66.3+ 10.4 (min=18, max=103)	0.399	67.6 + 10.8 (min=30, max=91)	66.3 + 10.4 (min=18, max=103)	0.7*
**Female (%)**	143 (46.9)	15893 (57.6)	<0.001	88 (59.1)	15948 (57.5)	0.92**
**Pre-surgery risk factors:**						
None (%)	125 (41.0)	14000 (50.8)	<0.001	44 (29.5)	14081 (50.8)	<0.001**
One (%)	74 (24.3)	6728 (24.4)		49 (32.9)	6753 (24.3)	
Two or more (%)	106 (34.8)	6859 (24.9)		56 (37.6)	6909 (24.9)	
**Procedure Type:**						
TKA (%)	146 (47.9)	16,239 (58.9)	< 0.001	90 (0.6)	16295 (99.4)	0.68**
THA, incl. hip resurfacing (%)	159 (52.1)	11348 (41.1)		59 (0.5)	11448 (99.5)	
**Procedure Duration:**						
<100 mins (%)	252 (88.7)	22892 (90)	0.475	118 (87.4)	23026 (90.0)	0.31**
≥100 mins (%)	32 (11.3)	2540 (10)		17 (12.6)	2555 (10.0)	

* t test for mean comparison. ** Chi-square test

**Table 4:** Unadjusted odds ratio (OR) between transfused and deep, superficial infection

	Transfused, N=3098	Non-Transfused, N=24798	Unadjusted OR [95% Confidence Interval]	p-value
**Deep infection**				
Yes (%)	51 (1.6)	254 (1)	1.6 [1.2 - 2.2]	0.002*
No (%)	3044 (98.4)	24,543 (99)		
**Superficial infection**				
Yes (%)	31 (1.0)	118 (0.5)	2.1 [1.4 - 3.1]	<0.001*
No (%)	3064 (99.0)	24,679 (99.5)		

* Logistic model using Wald's chi-squared statistic

**Table 5:** Multivariable logistic regression analysis for deep infection

Deep infection	Adjusted OR	p-value*	[95% Confidence Interval]
**Transfused vs. Non-transfused**	1.6	0.01	1.1 - 2.2
** TKA vs. THA**	0.6	<0.001	0.5 - 0.8
** Duration ≥100 vs. <100 mins**	1.0	0.84	0.7 - 1.5
** Age (>70 vs. ≤70)**	1.0	0.87	0.8 - 1.3
** Female vs. Male**	0.7	0.001	0.5 - 0.8
**Pre-surgery risk factors:**			
One vs. None	1.2	0.3	0.9 - 1.6
Two or more vs. None	1.7	<0.001	1.3 - 2.2

* Wald's chi-squared statistic. Area under ROC curve: 0.70

**Table 6:** Multivariable logistic regression analysis for superficial infection

Superficial infection	Adjusted OR	p-value*	[95% Confidence Interval]
**Transfused vs. Non-transfused**	1.9	0.005	1.2 - 2.9
**TKA vs. THA**	1.1	0.45	0.8 - 1.6
**Duration ≥100 vs. <100 mins**	1.2	0.49	0.7 - 2.0
**Age (>70 vs. ≤70)**	0.8	0.29	0.6 - 1.2
**Female vs. Male**	1.1	0.58	0.8 - 1.6
**Pre-surgery risk factors:**			
One vs. None	2.3	<0.001	1.5 - 3.6
Two or more vs. None	2.5	<0.001	1.6 - 3.8

* Wald's chi-squared statistic. Area under ROC curve: 0.68

**Table 7:** Provincial Blood Transfusion Guidelines

Scenario	Prior to Jan 1, 2015	After Jan 1, 2015
Hgb < 100 g/l & signs and symptoms of impaired O_2_ delivery, heart rate ≥ 100, systolic BP ≤ 90, respiratory ≥ 20, dyspnea, syncope, angina, confusion, ECG ischemic changes	O_2_, transfuse packed red blood cells 2 units and reassess	O_2_, transfuse packed red blood cells 1 unit and reassess
Hgb ≥ 70 g/l & no sign of impaired O_2_ delivery and no sign of cardiac history	Monitor	Monitor
Hgb < 70 g/l	Transfuse red blood cells 2 units and reassess	Transfuse red blood cells 1 unit and reassess

**Table 8:** Studies investigated the association between allogenic blood transfusion and SSI

Study	Year	Number of patients	Methodology	Type of joint replacement whether primary or revision	Postoperative Period considered for transfusion	Definition of infection and criteria for diagnosis	Follow-up in months	Association between blood transfusion and SSI
Al Mustafa et al (32)	2018	3932	retrospective cohort- Single center	Primary arthroplasty	Not reported	National Healthcare Safety Network SSI (CDC)	12	Positive association
Newman et al (33)	2014	3352	Retrospective	Primary arthroplasty	Not reported	Reoperation for clinically suspected infection	3	Positive association
Friedman et al (34)	2014	12177	Retrospective	Not reported	Not reported	Wound infection- bone and joint infection (no specific definition)	1.5-2	Positive association
Everhart et al (35)	2018	6788	Retrospective- single center	Primary and Revision arthroplasty	30 days after admission	Readmission with diagnosis of superficial or deep infection	12	Positive association
Kim et al (36)	2017	21770	Meta-analysis of 6 studies	Not reported	Not reported	Variable according to the study included	3-12 (3 studies did not report the FU duration)	Positive association
This study	2019	27892	Retrospective- Registry study (14 hospitals)	Primary hip and knee arthroplasty	During hospital stay	National Healthcare Safety Network SSI (CDC)	6	Positive association
